# CAPG is a novel biomarker for early gastric cancer and is involved in the Wnt/β-catenin signaling pathway

**DOI:** 10.1038/s41420-023-01767-6

**Published:** 2024-01-08

**Authors:** Yan Long, JiaQi Wu, Yu Shen, Chenxiao Gan, Chuandong Zhang, Gang Wang, Jiyong Jing, Chenjing Zhang, Wensheng Pan

**Affiliations:** 1 Cancer Center, Department of Gastroenterology, Zhejiang Provincial People’s Hospital, Affiliated People’s Hospital, Hangzhou Medical College, No. 158 Shangtang Road, Hangzhou, Zhejiang China; 2 Department of Gastroenterology, Affiliated Hospital of Hangzhou Normal University, 310015 Hangzhou, China; 3 Zhejiang Chinese Medical University, Hangzhou, China; 4 The Medical College of QingDao University, Qingdao, Shandong China; 5 Shulan International Medical College, Zhejiang Shuren University, Hangzhou, Zhejiang China; 6 Department of Medical Education and Simulation Center, Zhejiang Provincial People’s Hospital, Affiliated People’s Hospital, Hangzhou Medical College, 310014 Hangzhou, Zhejiang China

**Keywords:** Gastric cancer, Tumour biomarkers

## Abstract

Past studies have shown that the Gelsolin-like actin-capping protein (CAPG) regulates cell migration and proliferation and is strongly associated with tumor progression. We present the first study of the mechanism of action of CAPG in early gastric cancer (EGC). We demonstrate that CAPG expression is upregulated in gastric cancer (GC) especially EGC. CAPG promotes GC proliferation, migration, invasion, and metastasis in vivo and in vitro. More importantly, CAPG plays a role in GC by involving the Wnt/β-catenin signaling pathway. Our findings suggest that CAPG may function as a novel biomarker for EGC.

## Introduction

Gastric cancer (GC) is one of the most prevalent cancers worldwide, especially in East Asian countries [1, 2]. In China, there are an estimated 679,100 new cases and 498,000 deaths per year, ranking GC second among types of cancer in this population [2]. GC has no obvious clinical symptoms in its early stages and is diagnosed at an advanced stage. The WHO has clearly noted that early detection is the key to improving cancer treatment outcomes. When cancer is detected at the earliest stages, treatment is more effective, and survival drastically improves; though, early detection, early diagnosis, and early treatment of cancer can reduce the mortality rate [3]. For example, the 5-year survival rate for advanced gastric cancer (AGC) in most parts of the world is approximately 20%, but for early gastric cancer (EGC), it is greater than 90% [4, 5]. Therefore, finding new biomarkers for EGC, improving the detection rate, and fully elucidating the molecular mechanism of cancer metastasis would undoubtedly facilitate the development of targeted therapies and improve the prognosis of patients.

Gelsolin-like actin-capping protein (CAPG) is a member of the gelsolin superfamily that caps and prevents the growth of actin filaments in a Ca^2+^ and polyphosphatidylinositol-dependent manner but does not sever them [6, 7]. A series of investigations have shown that upregulation of CAPG has been observed in a variety of cancer cells, including breast, pancreatic, lung, liver, and prostate cancers [8–13], and CAPG promotes the proliferation, migration, invasion, and metastasis of a wide range of tumor cells [9, 10, 12, 14–22]. These studies suggest that CAPG appears to be a key player in different biological activities in cells and that CAPG may be a potential therapeutic target for GC.

The Wnt/β-catenin signaling pathway plays an important role in normal embryo development, tissue differentiation, homeostasis, and oncogenesis [23], and Wnt signaling regulates various functions of cancer cells, such as proliferation, cell fate determination, motility, and invasion, through activation of β-catenin [24]. Furthermore, more than half of GC patients have dysregulated Wnt/β-catenin signaling, a major cause of gastric cancer progression [23, 25], although the mechanism underlying abnormal β-catenin activation in GC is unclear.

In this study, we performed gel-based proteomics experiments to obtain differentially expressed proteins from a sufficiently large cohort of GC. Our mass spectrometry experiments were designed mainly to analyze the differential proteins between EGC and normal tissues and to explore new biomarkers for EGC. Analysis of the mass spectrometry results concluded that CAPG was highly expressed in EGCs. Therefore, we collected clinical specimens to validate CAPG expression in EGCs and evaluated the effect of CAPG on GC biological functions by using up-and-downregulation of CAPG in GC cell lines and establishing animal models, in addition to investigating the role of CAPG in GC molecular mechanisms. We present the first study of the mechanism of action of CAPG in GC.

## Results

### CAPG was differentially expressed in GC, especially in EGC, by 2D DIGE

**Fig. 1:**
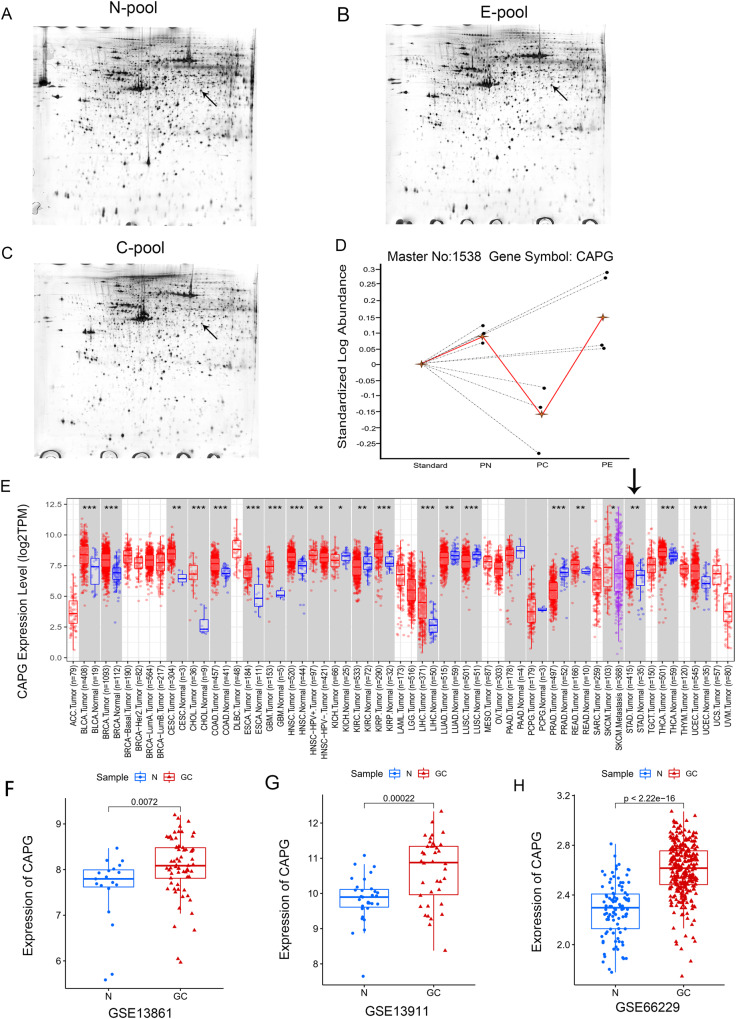
Bidirectional electrophoresis, mass spectrometry, and databases analysis of GC. **A**–**C** Bio-Rad GS 710 scanner (Bio-Rad) scan of simulated pool samples. **D** The relative abundance of CAPG. **E** Visualization of CAPG expression status in GC by TIMER2. **F**–**H** CAPG was differentially expressed in different GEO databases. E: early gastric cancer; N: normal; C: advanced gastric cancer; pool: equal mix of proteins from 3 low-differentiated cancer tissues.

**Fig. 2:**
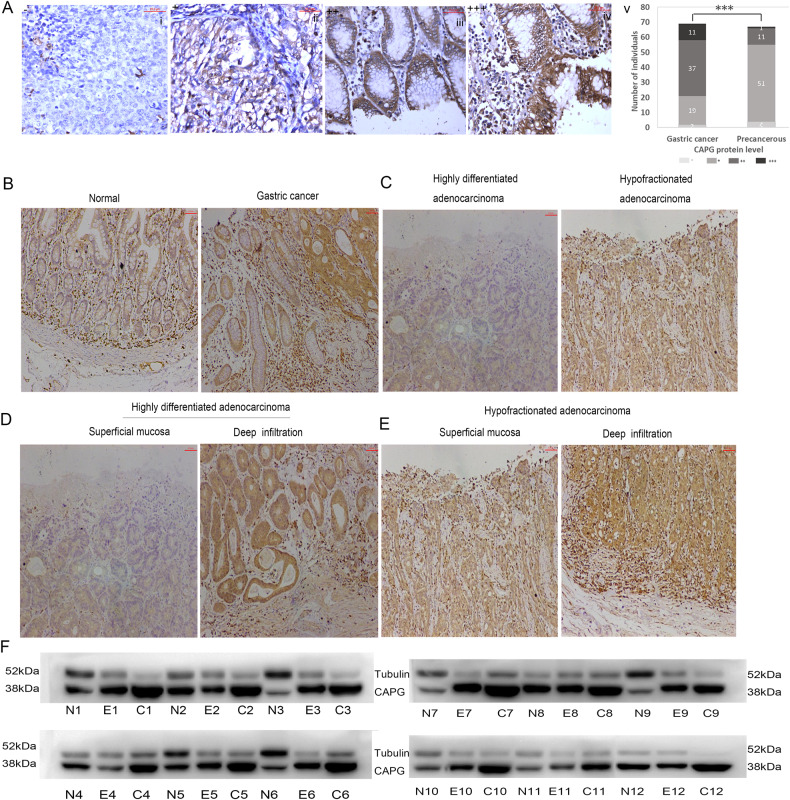
CAPG was upregulated in EGC. **A** IHC analysis of CAPG in GC, especially EGC, (i) Negative (CAPG–); (ii) Weak staining (CAPG+); (iii) Moderate staining (CAPG++); (iv) Strong staining (CAPG+++); (v) Bar chart shows the summary of the distribution of different CAPG protein levels in tumor for all GC (*n* = 69) and precancerous (*n* = 67). **B** CAPG is expressed in normal and GC, and CAPG is localized in the nucleus and cytoplasm. **C** CAPG is expressed in hypodifferentiated adenocarcinoma and highly differentiated adenocarcinoma. **D** CAPG is expressed in the deep infiltrated and superficial mucosa of highly differentiated adenocarcinoma. **E** CAPG is expressed in the deep infiltrated and superficial mucosa of hypodifferentiated adenocarcinoma. **F** Result of WB on twelve sets of clinical samples. N, normal; C, advanced gastric cancer; E, early gastric cancer.

According to 2D DIGE, the Bio-Rad GS 710 scanner (Bio-Rad) scans mimicked the gels (Fig. 1A–C). The aim of our study is mainly to explore new biomarkers of EGC, so the study focuses on the analysis of differential proteins between EGC and normal tissue. The abundance of differentially expressed proteins in EGCs after labeling and 3D simulations were used to show the relative abundance of CAPG (Fig. 1D). Analysis of the experimental results of 2D DIGE revealed that CAPG was differentially expressed in GC, especially in EGC. Then, we also analyzed whether CAPG was differentially expressed in GC in multiple databases. CAPG mRNA was significantly higher in gastric adenocarcinoma (STAD) (*p* < 0.01) (Fig. 1E), and CAPG was higher in GC than in normal tissues in GSE13861 (*p* = 0.0072; Fig. 1F), GSE13911 (*p* = 0.00022; Fig. 1G), and GSE66229 (*p* < 0.001; Fig. 2H). Multiple databases and 2D DIGE yielded concordant results that CAPG was differentially expressed in GC. More importantly, 2D DIGE showed that CAPG was upregulated in EGCs.

### CAPG was upregulated in EGC by IHC and WB, and CAPG was negatively correlated with lymph node metastasis

To determine CAPG protein expression in GC, we performed IHC staining for CAPG on 69 GC specimens and 67 precancerous specimens, showing representative images of IHC staining with different levels of CAPG expression (Fig. 2Ai–iv). Statistical analysis confirmed the upregulation of CAPG in malignant samples: 69.6% of GC (48/69) showed high expression, of which almost one-fifth (11/69, 16.0%) showed extensive and intense staining (CAPG +++) (Fig. 2Av). CAPG was mainly located in the cytoplasm, and its expression was significantly higher in GC than in normal tissues (Fig. 2B). CAPG was higher in hypofractionated adenocarcinoma than in highly differentiated adenocarcinoma (Fig. 2C). CAPG was stronger in the deep infiltrating than in the superficial mucosa of highly differentiated adenocarcinoma (Fig. 2D) and CAPG was stronger in the deep infiltrated than superficial mucosa of hypodifferentiated adenocarcinoma (Fig. 2E). Twelve sets of clinical samples were randomly selected for WB, and these results showed that CAPG was upregulated in GC (Fig. 2F). In addition, high CAPG was 69.6% (48/69) in GC and 17.9% (12/67) in precancerous lesions (Table 1). Interestingly, we found that CAPG was higher in EGC than in AGC (*p* = 0.005), and high CAPG was 94.7% (18/19) in EGC and 60% (30/50) in AGC (Table 1). In addition, CAPG was strongly correlated with lymph node metastasis, and CAPG expression was higher in tissues without lymph node metastasis (*p* = 0.00002) (Table 1). However, CAPG did not correlate with age, sex, or cancer differentiation (Table 1).

**Table 1:** Relationship between CAPG and clinical characteristics of GC.

Clinical characteristics	*N*	CAPG protein expression			Rate of high expression (%)			*p*-value
		Low		High				
Age (years)								
<60	21		5	16		76.2	0.341	
≥60	48		17	31		64.6		
Sex								
Male	50		15	35		70.0	0.899	
Female	19		6	13		68.4		
Differentiation								
Poor	52		17	35		67.3	0.476	
Well	17		4	13		76.5		
Group								
Precancerous	67		55	12		17.9	*p* < 0.0001***	
GC	69		21	48		69.6		
Clinical stage								
EGC	19		1	18		94.7	0.005**	
AGC	50		20	30		60		
Lymph node metastases								
Negative	50		8	42		84	0.00002***	
Positive	19		13	6		31.6		

*CAPG* gelsolin-like actin-capping protein, *EGC* early gastric cancer, *AGC* advanced gastric cancer, *GC* gastric cancer.

^*^p < 0.05, **p < 0.01, ***p < 0.001; *p*-value was calculated by a Chi-square test.

In conclusion, the results of IHC and WB on clinical samples showed that CAPG was upregulated in EGC and negatively correlated with lymph node metastasis.

### CAPG is an unfavorable prognostic factor for GC

**Fig. 3:**
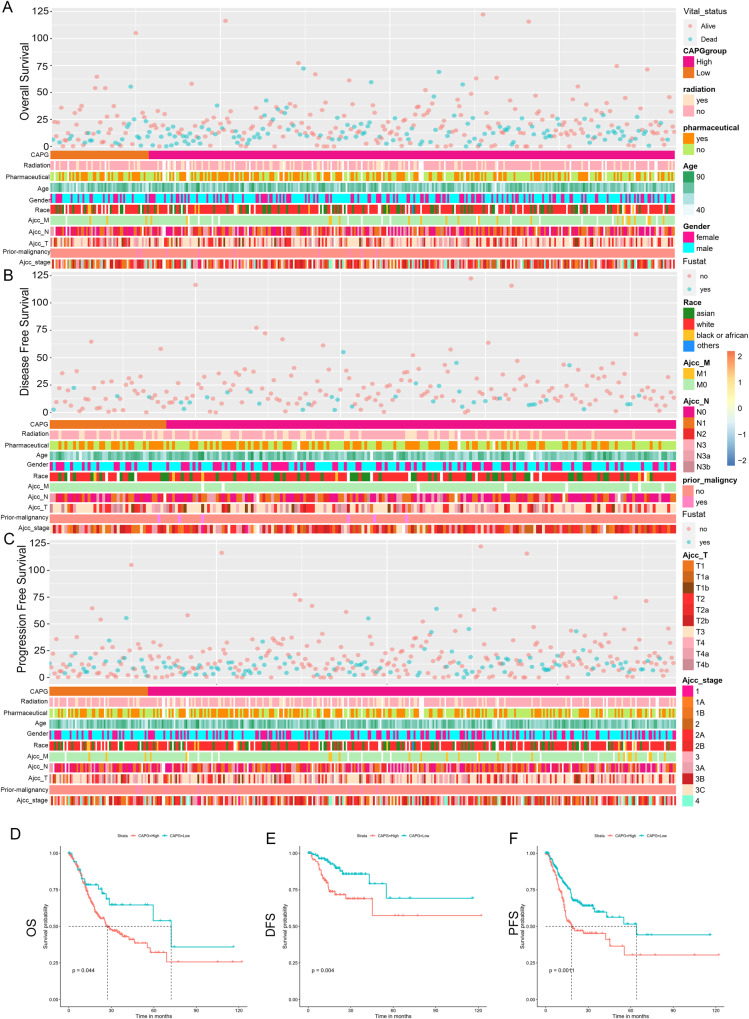
CAPG is an unfavorable prognostic factor for GC. Correlation between clinical characteristics and OS (**A**), DFS (**B**), and PFS (**C**) for GC in the TCGA database. Kaplan–Meier analysis of CAPG in TCGA database (**D**–**F**). The 95% confidence intervals for the high and low CAPG groups are indicated by the red and blue dashed lines, respectively. CAPG gelsolin-like actin-capping protein, OS overall survival, DFS disease-free survival, PFS progression-free survival, TCGA the Cancer Genome Atlas, GC gastric cancer.

We investigated the correlation between CAPG and the prognosis of GC based on the TCGA database. CAPG was divided into high CAPG and low CAPG groups according to X-tile software (Yale University, New Haven, CT, USA), and the software was used to determine the optimal cut-off value. Patients with different levels of CAPG showed different patterns of clinical and pathological characteristics. The correlation between CAPG and prognostic of GC was analyzed by clinical dates and mRNA of CAPG downloaded from TCGA (*n* = 433), and the relationship between clinical indexes and overall survival (OS), disease-free survival (DFS), and progression-free survival (PFS) of GC are shown in Fig. 3A–C. These plots showed that CAPG was enriched in patients with high malignancy of GC. To investigate the prognostic value of CAPG in GC, we performed Kaplan‒Meier and Cox proportional risk model analyses based on the TCGA database. In the TCGA database, patients with high CAPG had significantly shorter OS (*p* = 0.044), DFS (*p* = 0.004), and PFS (*p* = 0.0011) than patients with low CAPG (Fig. 3D–F). CAPG was further validated as an independent prognostic factor for OS in GC in a one-way multifactorial Cox regression analysis [hazard ratio (HR) = 1.752, 95% confidence interval (CI): 1.013–3.029, *p* = 0.045] (Table 2). In summary, CAPG is an unfavorably prognostic factor for GC.

**Table 2:** Univariate and multivariate OS analysis of various prognostic factors for GC by the Cox proportional hazard model.

Variables	Univariate analysis		Multivariate analysis	
			Hazard ratio for OS (95% CI)	*p*-value
	Hazard ratio for OS (95% CI)	*p*-value		
Age	1.022 (1.005–1.039)	0.01**	1.028 (1.009–1.047)	0.003**
Sex	1.282 (0.899–1.83)	0.171		
Pathologic stage (I/I vs. III/IV)	1.94 (1.352–2.785)	<0.001***	1.436 (0.82–2.517)	0.206
Pathologic N (N0 vs. N1,2,3)	1.005 (1.002–1.009)	0.002**	1.001 (0.994–1.008)	0.758
Pathologic M (M0 vs. *M1)*	2.277 (1.283–4.041)	0.005**	2.227 (1.176–4.217)	0.014*
CAPG expression (low vs. high)	1.68 (1.009–2.797)	0.046*	1.752 (1.013–3.029)	0.045*

*CI* confidence interval, *OS* overall survival.

^*^p < 0.05, **p < 0.01, ***p < 0.001.

### CAPG promotes GC proliferation in vivo and in vitro

**Fig. 4:**
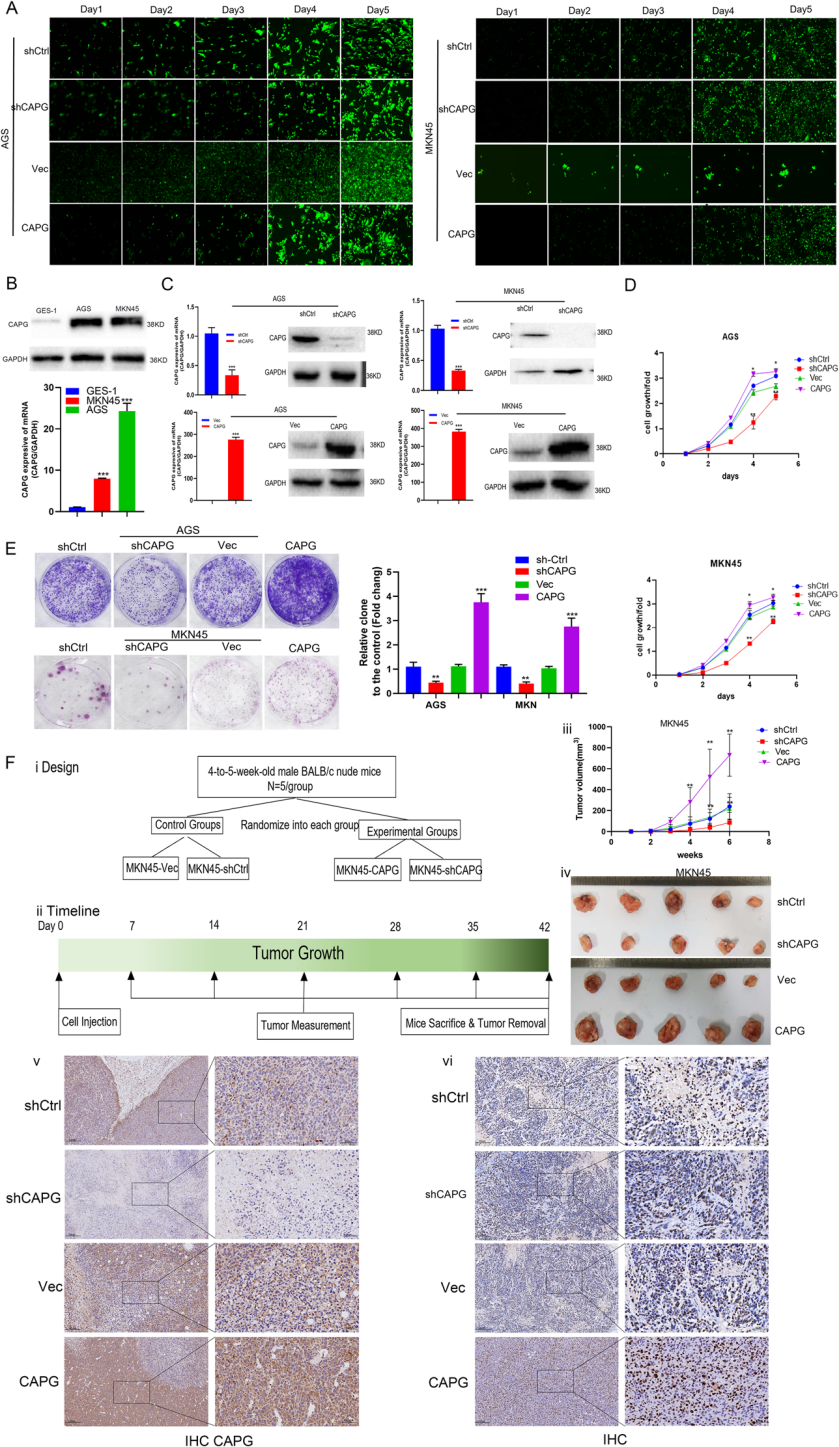
CAPG promotes GC proliferation in vivo and in vitro. **A** Representative fluorescent images of AGS and MKN45 cells at different time points (days 1–5) displayed by Celigo Imaging cytometry. **B** CAPG protein and mRNA expression were upregulated in MKN45 and AGS than GES-1. **C** Upregulation and downregulation of CAPG efficiency in GC cells were detected by qRT-PCR and WB, with GAPDH as a loading control. **D** Cell growth curves showed that upregulation of CAPG increased the proliferation of MKN45 and AGS cells, but downregulation of CAPG attenuated the proliferation. **E** Representative images and statistical summary plots show that the upregulation of CAPG enhanced the colony-forming ability of MKN45 and AGS cells, while the downregulation of CAPG attenuated colony-forming ability, and quantitative analysis of the number of foci is shown in the right panel. **F** i and ii. Timeline plots of in vivo tumorigenesis study design and xenograft experiments. iii. Tumor growth curves are summarized in line graphs. iv. Representative pictures of subcutaneous tumors formed in nude mice after injection of MKN45-shCtrl, MKN45-shCAPG, MKN45-Vec, and MKN45-CAPG cells and each group includes 5 mice. v and vi. IHC staining of sectioned tissue with anti-CAPG and anti-ki67 antibodies (left: x100 magnification; right: x400 magnification). *p < 0.05, **p < 0.01, ***p < 0.001, results represent the mean ± SD of three independent experiments. CAPG gelsolin-like actin-capping protein, IHC immunohistochemistry, qRT-PCR quantitative real-time polymerase chain reaction, SD standard deviation, shCtrl control cells, Vec vector, GAPDH glyceraldehyde 3-phosphate dehydrogenase.

Furthermore, some experiments were designed to investigate the role of CAPG in GC. These experimental results showed that upregulation of CAPG promotes GC cell proliferation (^*^p < 0.05, **p < 0.01; Fig. 4D). Similarly, upregulation of CAPG produced more foci in the foci formation assay (**p < 0.01, ***p < 0.001; Fig. 4E). Consistent with the in vitro results, upregulation of CAPG promoted tumor volume growth, as evidenced by the final tumor volumes and tumor growth curves (^*^p < 0.05, **p < 0.01; Fig. 4Fiii–iv). IHC staining of tumors confirmed that upregulation of CAPG enhanced the expression of CAPG and ki67 in tumors (Fig. 4Fv–vi). In contrast, the downregulation of CAPG showed the opposite effect both in vitro and in vivo. Thus, these data suggest that CAPG promotes GC proliferation in vivo and in vitro.

### CAPG promotes GC migration, invasion, and metastasis in vivo and in vitro

**Fig. 5:**
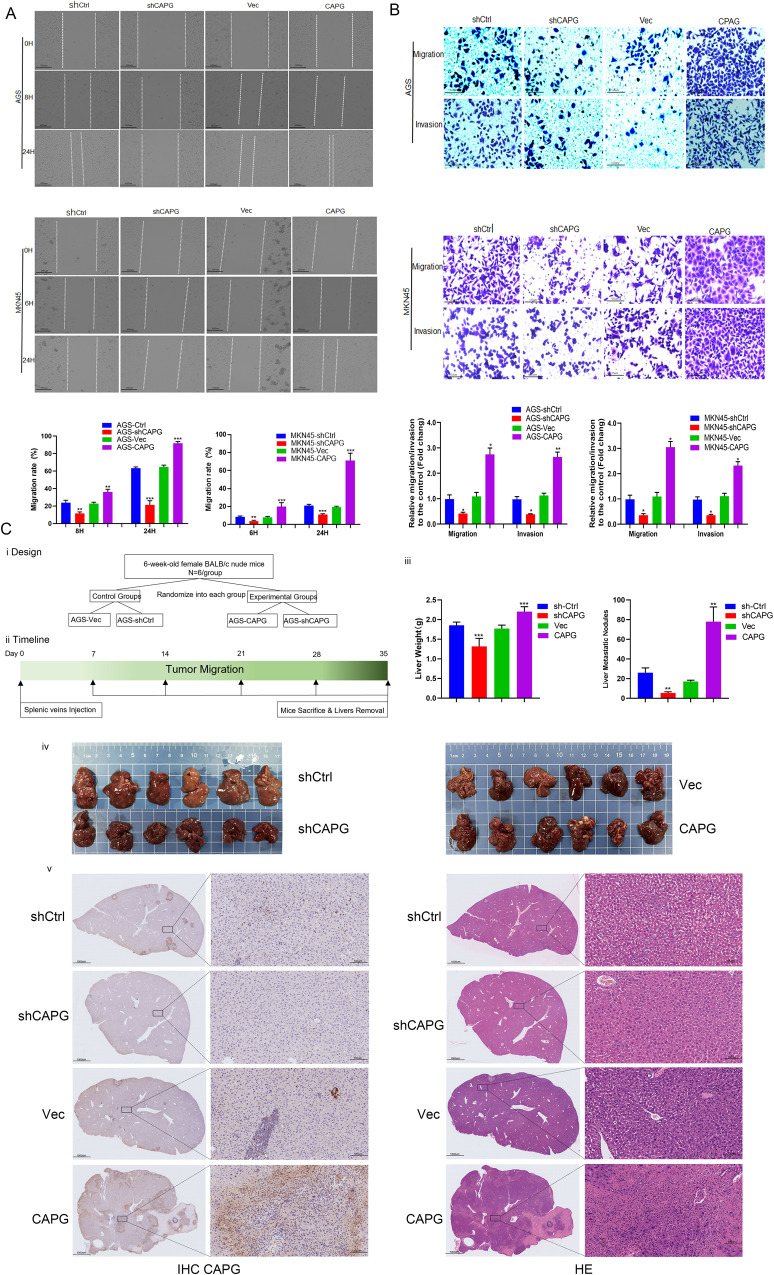
CAPG promotes migration, invasion, and metastasis in GC. **A** Representative images (top) and quantification (bottom) of wound healing experiments and migration rate were calculated as the ratio of migration distance to wound gap at 0 h. Cell migration distance at 0 h was set to 0. **B** Representative microscopic views of MKN45 and AGS cells migrating through transwell and invading matrigel membranes taken under the microscope after 24 h of incubation (top), and graphs (bottom) showing folded migrating and invading cell numbers compared to control changes. The mean ± SD is shown for three independent experiments performed in triplicate for each group. Statistical significance was measured by Student’s t-test., *p < 0.05, **p < 0.01, ***p < 0.001. **C** CAPG enhances GC metastasis in a splenic vein mouse metastasis model. i. In vivo metastasis study design. ii. Timeline graph of the experiment, 35 days after the liver of mice was detached from the surrounding tissue. The data represent the mean ± SD of 6 mice per group, **p < 0.01, ***p < 0.001 by Student’s t-test. iii. The measurements of liver weight and tumor nodules were counted, and the data represent the mean ± SD of 6 mice per group; **p < 0.01, ***p < 0.001 by Student’s *t*-test. v. IHC staining of liver sections with CAPG antibody (left panel at ×1 magnification, right panel at ×100 magnification). CAPG gelsolin-like actin-capping protein, IHC immunohistochemistry, SD standard deviation, shCtrl control cells, GC gastric cancer, Vec vector.

We designed experiments to investigate whether CAPG influences GC cell migration, invasion, and metastasis. After establishing the stable expression of cells, wound healing assays showed that downregulation of CAPG delayed the spread of GC cell lines, and upregulation of CAPG accelerated the spread of MKN45 and AGS cells. Quantitative analysis of wound healing is shown in the Fig. 5A (*p < 0.05, **p < 0.01). Further migration assays showed that downregulation of CAPG attenuated the migration ability of MKN45 and AGS cells, and upregulation of CAPG enhanced the migration ability of MKN45 and AGS cells. Quantitative analysis of migration is shown in Fig. 5B. Similarly, downregulation of CAPG decreased the invasiveness of MKN45 and AGS cells, and upregulation of CAPG increased the invasiveness of MKN45 and AGS cells. Quantitative analysis of invasion is shown in Fig. 5B (*p < 0.05, **p < 0.01). These results suggest that the upregulation of CAPG significantly enhances cancer migration and invasion in GC and vice versa. Consistent with the in vivo results, upregulation of CAPG resulted in more pulmonary liver weight and liver nodules at 35 days postinjection than in the controls while downregulation of CAPG resulted in lower pulmonary liver weight and liver nodules than in the controls, implying that CAPG is essential for GC cell colonization in the liver (**p < 0.01, ***p < 0.001; Fig. 5Ciii). IHC and HE analysis showed that upregulation of CAPG formed many metastatic colonies, while downregulation of CAPG formed fewer metastatic colonies compared to controls (Fig. 5Cv). Our data suggest that CAPG promotes GC migration, invasion, and metastasis in vivo and in vitro.

### CAPG is involved in the Wnt/β-catenin signaling pathway

**Fig. 6:**
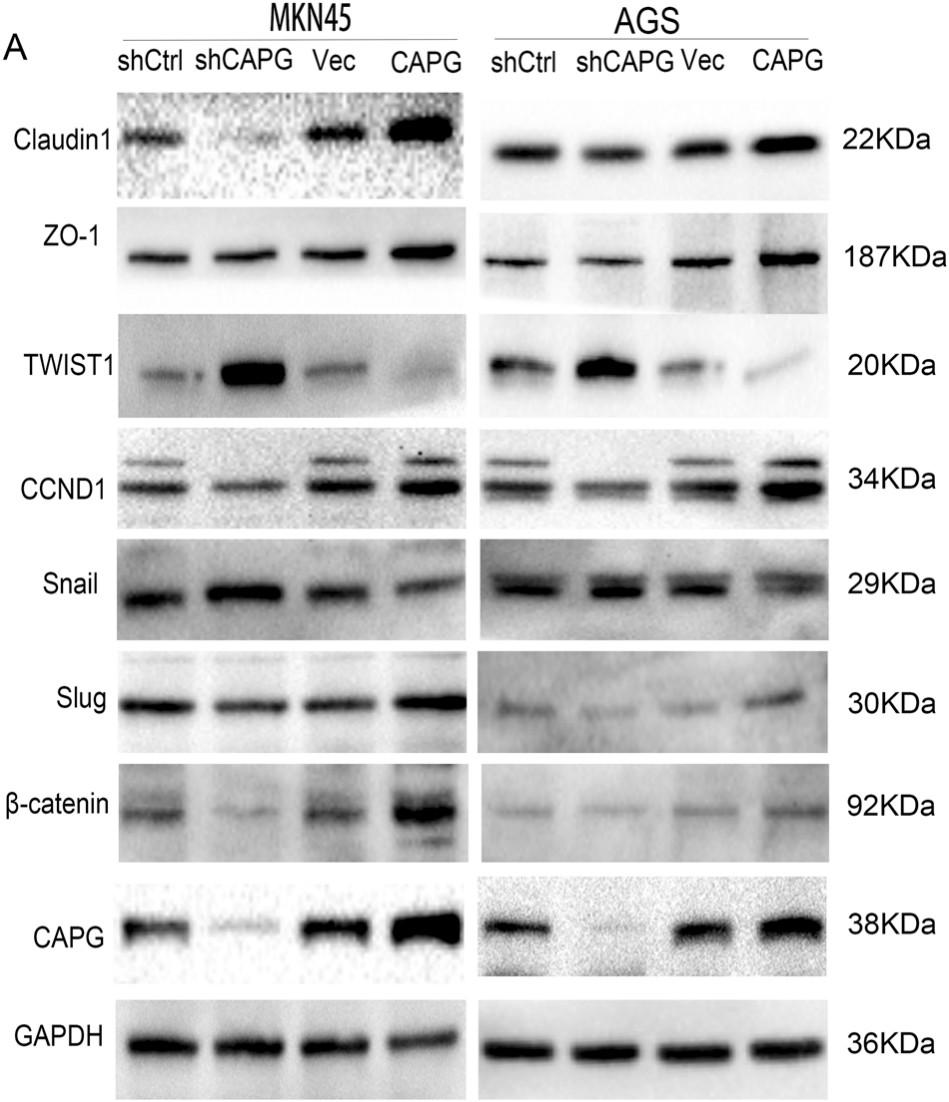
CAPG is involved in the Wnt/β-catenin signaling pathway. **A** Western blot analysis. CAPG, gelsolin-like actin-capping protein; shCtrl, control cells; Vec, vector.

**Fig. 7:**
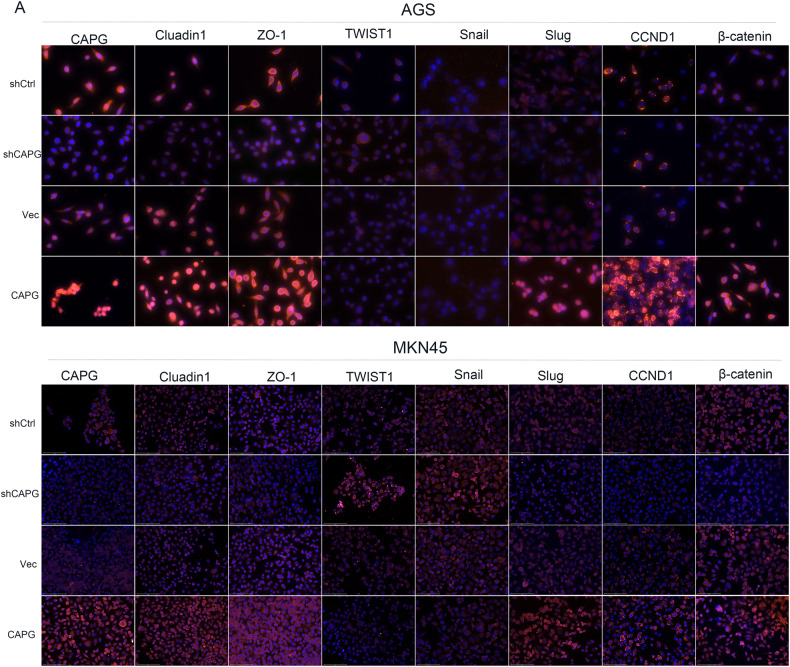
CAPG is involved in the Wnt/β-catenin signaling pathway. **A** Immunofluorescence analysis. CAPG, gelsolin-like actin-capping protein; shCtrl control cells, Vec vector.

Further experiments were conducted to explore a signaling pathway by which CAPG might play a role in GC. The Wnt/β-catenin signaling pathway plays a crucial role in GC [26]. So, we needed to verify whether CAPG plays a crucial role in GC by involving in the Wnt/β-catenin signaling pathway. So, we performed WB analysis of key proteins involved in the Wnt/β-catenin signaling pathway, such as β-catenin, CCND1, Snail, Slug, ZO-1, Cluadin1, and TWIST1, in CAPG-overexpressing and CAPG-silenced GC cells. The β-catenin, CCND1, ZO-1, Cluadin1 and Slug protein levels were significantly reduced, but the TWIST1 and Snail protein levels were significantly increased in the CAPG-silenced cells, and the opposite results were obtained when CAPG was overexpressed in AGS and MKN45 cells (Fig. 6A). In addition, the opposite result was obtained after upregulation of CAPG in AGS and MKN45 cells. Furthermore, the experimental results obtained by IF were consistent with those obtained by WB (Fig. 7A). These results suggest that CAPG is involved in the Wnt/β-catenin signaling pathway.

These findings suggest that CAPG plays a role in GC by involving in the Wnt/β-catenin signaling pathway.

## Discussion

GC is the third most common cause of cancer-related death worldwide and the most common gastrointestinal malignancy in China [27–29]. Uncontrolled proliferation is the main characteristic of GC and poses a threat to human health. As a member of the gelsolin family, CAPG performs important biological and physiological functions in a variety of tissues [21].

Upregulation of CAPG has been observed in a variety of cancer tissues, including prostate, ovarian, pancreatic, and breast tissues [9, 10, 12, 30], and our study confirmed that CAPG expression was upregulated in GC. Patient follow-up has shown that enhanced CAPG levels are associated with shorter survival times and that CAPG can be considered an independent predictor of prognosis [9, 30, 31]. Our study confirmed that CAPG is an unfavorable prognostic factor for GC and that OS, DFS, and PFS were shortened when CAPG expression was upregulated. Our results showing that CAPG can promote GC proliferation are consistent with the results of a study showing that CAPG can promote prostate cancer proliferation [9]. However, the finding that CAPG does not promote GC proliferation [17] is inconsistent with other results, and this may be a discrepancy caused by the inconsistency of the applied cell lines. CAPG promotes GC invasion, migration, and metastasis, consistent with the findings of Ichikawa et al. [17]. In addition, CAPG promotes the migration, invasion, and metastasis of a variety of tumors [12, 14–16, 18, 20]. As a prerequisite for tumor cell invasion and metastasis, CAPG can mediate cell migration in both normal and cancer cells [32]. Due to the lack of cytoplasmic CAPG protein, cells are unable to properly reorganize their actin cytoskeleton during motility, possibly due to inappropriate elongation of actin filaments. Furthermore, the Wnt/β-catenin protein signaling pathway regulates metastasis in GC [26]. The Wnt/β-catenin signaling pathway, also called the canonical Wnt signaling pathway, is a conserved signaling axis that participates in diverse physiological processes, such as proliferation, differentiation, apoptosis, migration, invasion, and tissue homeostasis [33–35]. Furthermore, our study concludes that CAPG acts in GC by being involved in the Wnt/β-catenin signaling pathway. A previous study showed that CAPG promotes invasion and metastasis in GC, particularly through the invasion of lymphatic vessel components [17]. However, our findings showed that CAPG was negatively correlated with lymph node metastasis. This difference may be due to individual differences in the samples, and the previous study did not perform an IHC analysis. The number of samples collected in this study is limited, and a large-scale investigation is needed to elucidate the potential role of CAPG in EGC.

In conclusion, this is the first study of the role of CAPG in EGC. We demonstrated that CAPG is upregulated in EGC, especially in samples without lymph node metastasis, and that CAPG promotes GC proliferation, migration, invasion, and metastasis in vivo and in vitro. More importantly, CAPG plays a role in GC by being involved in the Wnt/β-catenin signaling pathway. Our findings suggest that CAPG may act as a novel biomarker for EGC.

## Materials and methods

### Big data analysis

We used several GEO (https://www.ncbi.nlm.nih.gov/geo/) datasets, including GSE13861, GSE13911, and GSE66229 and the Tumor Immune Estimation Resource version 2 (TIMER2) (https://timer.cistrome.org/).

### Gastric cancer specimens

Sixty-nine GC patients were obtained from the Zhejiang Provincial People’s Hospital and approved by the Institutional Review Board of the Zhejiang Provincial People’s Hospital (approval number: QT2023236).

### Cell culture and transfection

GC cell lines, AGS and MKN45, were purchased from the Shanghai Institute of Biochemistry and Cell Biology and cultured in RPMI1640 supplemented with 10% fetal bovine serum (FBS) and 1% penicillin/streptomycin. Cell plates were placed at 37 °C in a fully humidified incubator of 5% CO_2_ in air.

### Animals

The experimental protocol was approved by the Animal Welfare Committee of Zhejiang Provincial People’s Hospital (approval number: A20230001). All the mice were kept in standard cages at 26 ± 1°C under a 12/12 h light/dark cycle and fed the rodent standard diet with free access to water.

For the in vivo tumorigenic study, we used 4–5-week-old male BALB/c nude mice, Control cells (MKN45-Vec, MKN45-shCtrl), and CAPG-overexpressing or CAPG-knockdown cells (MKN45-CAPG, MKN45-shCAPG) were subcutaneously injected into the right dorsal flank of nude mice in a laminar flow cabinet. The cell number injected into each mouse was 5 × 10^6^, which were suspended in 100 µL PBS. The length (L) and width (W) of tumors were measured by calipers once every week and tumor volume was calculated by the formula *V* = 0.5 × *L* × *W*^2^ [36].

For the metastatic model, a total of 2 × 10^6^ AGS-CAPG cells, AGS-shCAPG, AGS-shCtrl or AGS-Vec cells were suspended in 50 µL sterile PBS and then injected into the spleens of 6-week-old female BALB/c nude mice. 2 × 10^6^ AGS cells were suspended in 50 μL sterile PBS and injected into the spleens of 6-week-old female BALB/c nude mice. A total of six mice were used for each group. After 5 weeks from the cell injection, mice were sacrificed, and the livers were collected. The weight of the livers and visible metastatic tumor nodules on the surface of the livers were quantified.

### CAPG overexpression and knockdown

The full-length wildtype CAPG cDNA was cloned into the LV5(EF-1a/GFP&Puro) expression vector. Cells transfected with the empty vector were used as a control (Vec). To effectively suppress CAPG expression, a small interfering RNA (sequence: 5’-GCTGATATCTGATGACTGCTT-3’) specifically targeting CAPG was designed and transformed into short hairpin RNA (shCAPG). The sequence of the scrambled shRNA (5’-TTCTCCGAACGTGTCACGTAA-3’) was used as a negative control (shCtrl). Stable clonal cell lines were selected using puromycin (Sigma-Aldrich) after transferring the lentivirus into the cells.

### Quantitative real-time polymerase chain reaction

Total RNA was extracted from the cells using RNA-Quik Purification Reagent (AG Biotechnology, China) according to the manufacturer’s procedures. The cDNA was reverse transcribed using the Reverse Transcription Polymerase Chain Reaction (RT-PCR) Kit (AG Biotechnology, China). Quantitative RT-PCR was performed in triplicate using the SYBR Green Premix Pro TaqHS qPCR kit (AG Biotechnology, China). Glyceraldehyde 3-phosphate dehydrogenase (GAPDH) expression was used as an internal control. Primers were synthesized by Invitrogen (CAPG, 5′- AGTCAGCATTTCACAAGACCTC-3′ and 5′-CAGGCGAAGATGTTCTGGC-3′; GAPDH, 5′-GGAGCGAGATCCCTCCAAAAT-3′ and 5′-GGCTGTTGTCATACTTCTCATGG-3′; forward and reverse). All results are expressed as fold change in mRNA expression relative to control cells and data are interpreted using 2^−∆∆Ct^.

### Western blot

Cells and tissues were lysed, and the proteins were collected. Next, the protein samples were separated and moved to PVDF membranes. After that, the membranes sealed by non‐fat milk were incubated with primary antibodies. Later, a secondary antibody was added. Anti-CAPG (ab155688), anti-GAPDH (ab16891), anti-β-catenin (ab32572), anti-N-cadherin (ab76011), anti-E-cadherin (ab40772), anti-CCND1 (ab16663), anti Cluadin1 (ab180151), anti-ZO-1(ab307799) purchased from Abcam, anti-Snail(A5243), anti-Slug (A1057), purchased from ABclonal.

### Immunofluorescence

The immunofluorescence (IF) staining was performed on GC cells. Briefly, cells were spread on cell crawls and washed three times in PBS, then fixed in 4% paraformaldehyde for 10 min, washed three times in PBS and permeabilized in 0.2% Triton X-100 for 10 min, washed three times in PBS, and closed with 5% bovine serum albumin for 30 min at room temperature, washed three times in PBS and then Cells were incubated with primary antibody at 4 °C overnight. After washing three times with PBS the cell crawls were incubated in a dark humidified chamber for 1 h at room temperature with the secondary antibody.

### Immunohistochemistry

The immunohistochemistry (IHC) procedure and scoring of CAPG expression were performed as per the standard protocol [37]. A total of 69 tissue samples were stained using anti-CAPG antibody. The intensity of CAPG expression was scored as follows: negative = 0, weak = 1, moderate = 2, strong = 3. The degree of staining was classified according to the percentage of positive cells, and they were scored as follows: negative = 0, 1% to 25% = 1, 26% to 50% = 2, 51% to 75% = 3, 76% to 100% = 4. The final grade for each gastric and precancerous tissue was calculated by multiplying the two scores. A score <6 indicates a low expression of CAPG and a score ≥6 indicates a high expression of CAPG. ALL scores were independently assessed by two pathologists who kept all clinical data confidential.

### CCK-8 assay

A suspension of 100 μL of medium containing 2000 cells per well was spread in 96-well plates 1 day prior to the assay. Cell viability was assayed using the CCK-8 kit according to the manufacturer’s instructions. Overall, three independent experiments were performed.

### Cell migration and invasion assays

Briefly, cells were cultured in six-well plates. Twenty-four hours before wound scratching, the complete medium was replaced with a serum-free medium. After scratching with a sterile tip, the medium was replaced to remove cell debris. A series of photographs were taken at different times. In addition, migration and invasion assays were performed in a 24-well. Briefly, cells were plated at a density of 3 × 10^4^ cells per well in the upper chamber. Cells were incubated for 1 h at 37 °C in 200 μL serum-free medium. 700 μL of medium containing 20% FBS was then added to the lower layer, followed by incubation at 37 °C for 24 h. Cells penetrating Matrigel were fixed, crystal violet stained, counted and photographed using fluorescence microscopy.

### Colony formation

For the colony formation assay, 1000 cells were seeded in 6-well plates and incubated at 37 °C for 14 days. Cells were then fixed in 4% paraformaldehyde for 20 min and stained with crystal violet solution for 15 min. Colonies (>50 cells/colony) were counted for the former.

### 2D gel electrophoresis

As described by Marouga et al. [38] and Nibbe et al. [39]. Outlining our overall experimental designs and emphasizing the use of an integrated histological approach to understand the pathophysiology of EGC. We first performed a quantitative proteomic analysis of the EGC cohort. (Fig. steps 1–11).
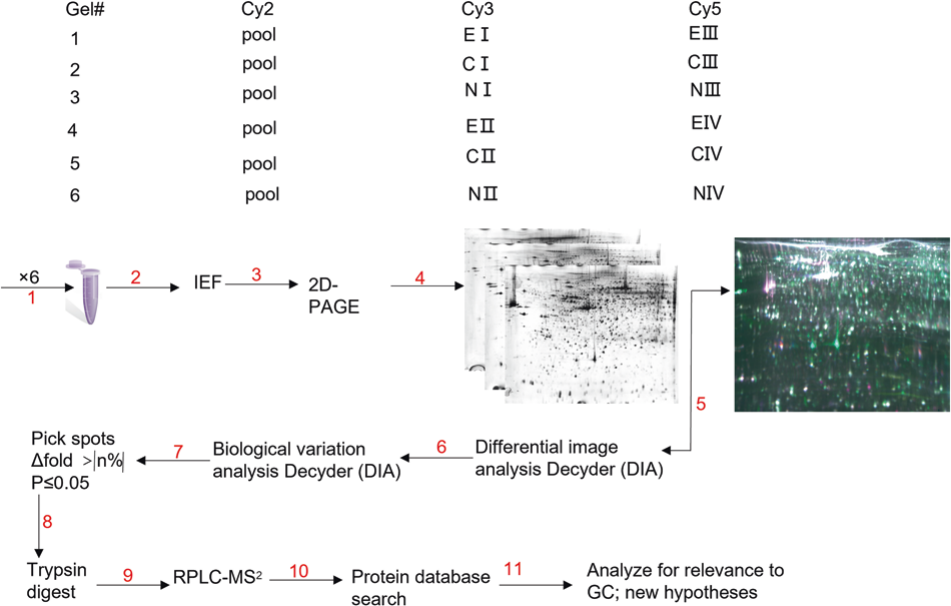


### Statistical analysis

The study was statistically analyzed using BM SPSS Statistics28 software and GraphPad Prism 9.2.0. Data were expressed as mean ± standard deviation (SD) of three independent experiments. Differences were considered statistically significant at *p* < 0.05, with *p*-values expressed as *p < 0.05, **p < 0.01, ***p < 0.001 in all figures.

### Supplementary information

Western blot

## Data Availability

All data are available upon request.
