# Supplementary figures and images for: CAPG is a novel biomarker for early gastric cancer and is involved in the Wnt/β-catenin signaling pathway
Source: Cell Death Discov. 2024 Jan 8;10:15. doi: 10.1038/s41420-023-01767-6 (PMC10774411; doi:10.1038/s41420-023-01767-6)

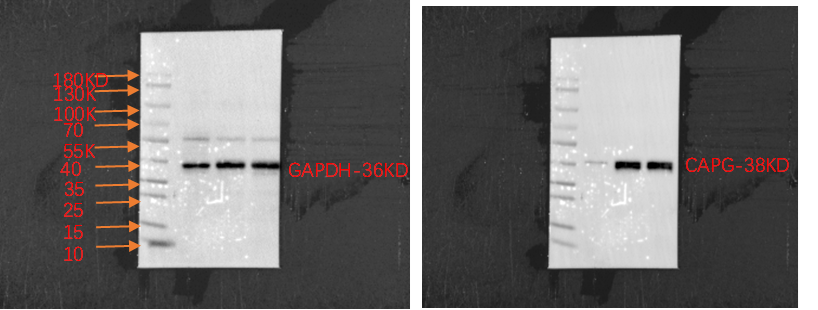


**Wnt/β-catenin signalling pathway**


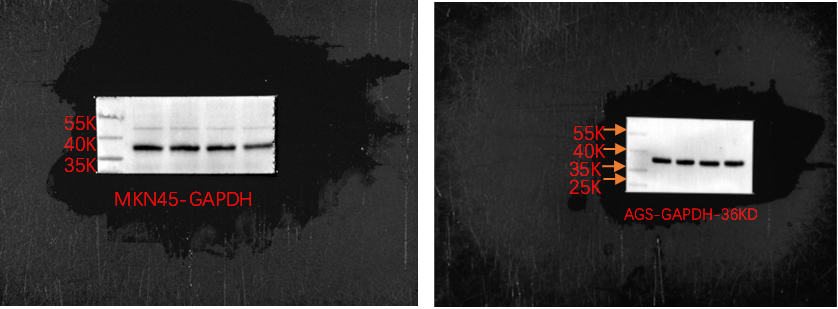


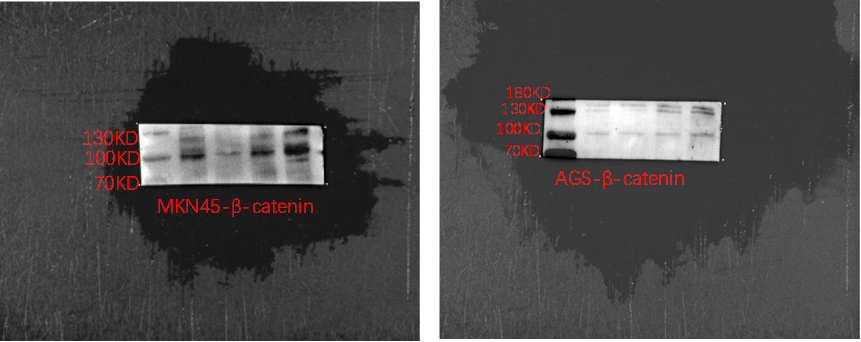

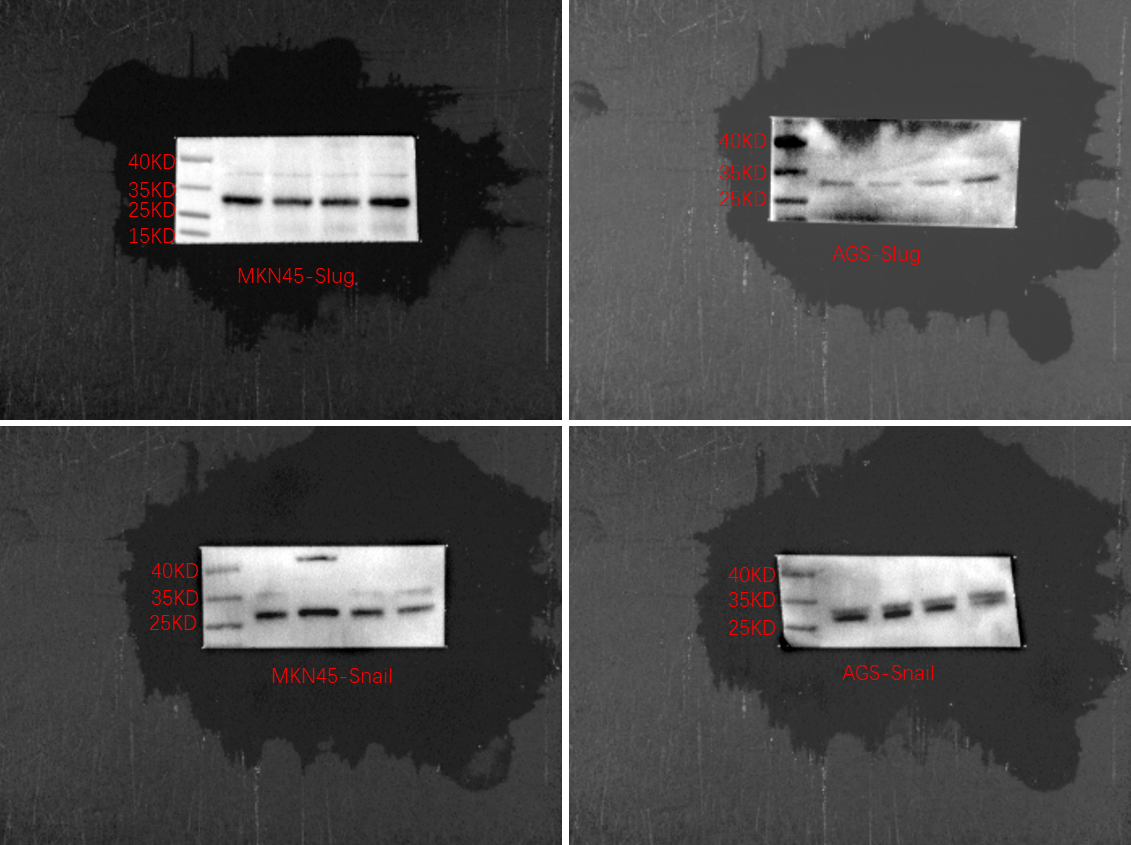

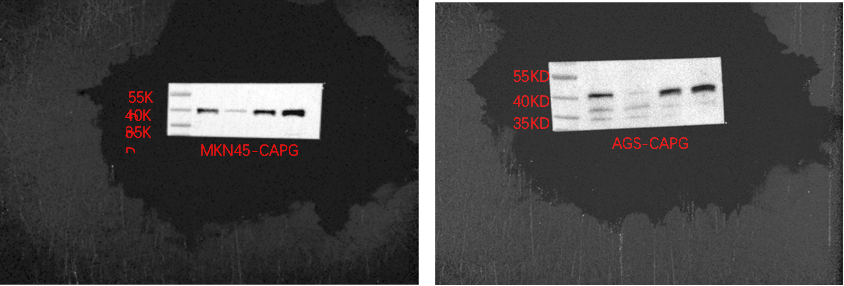


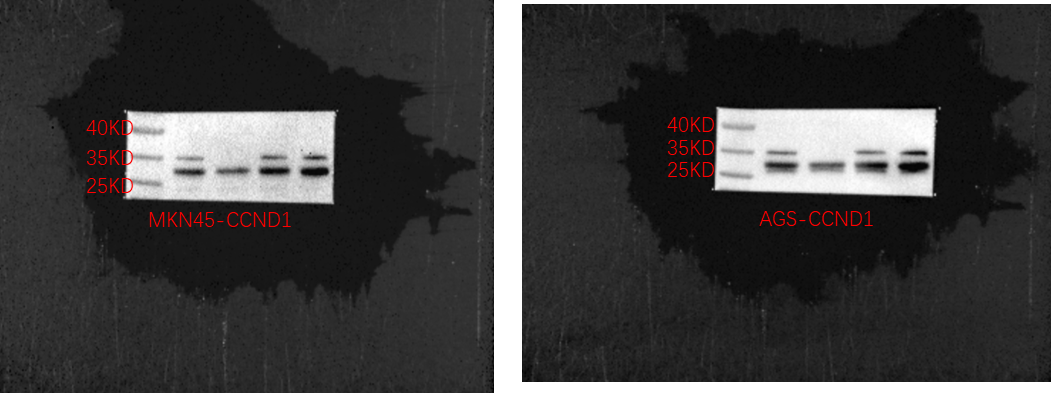


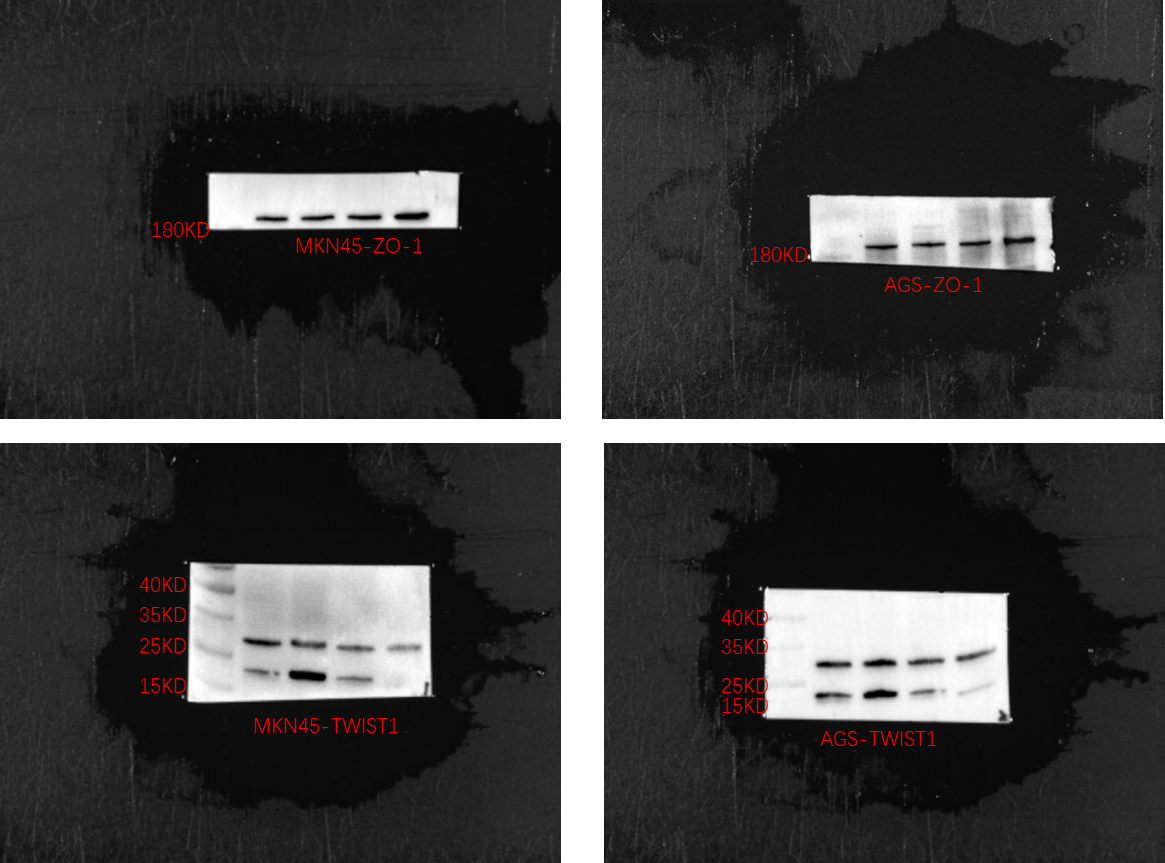

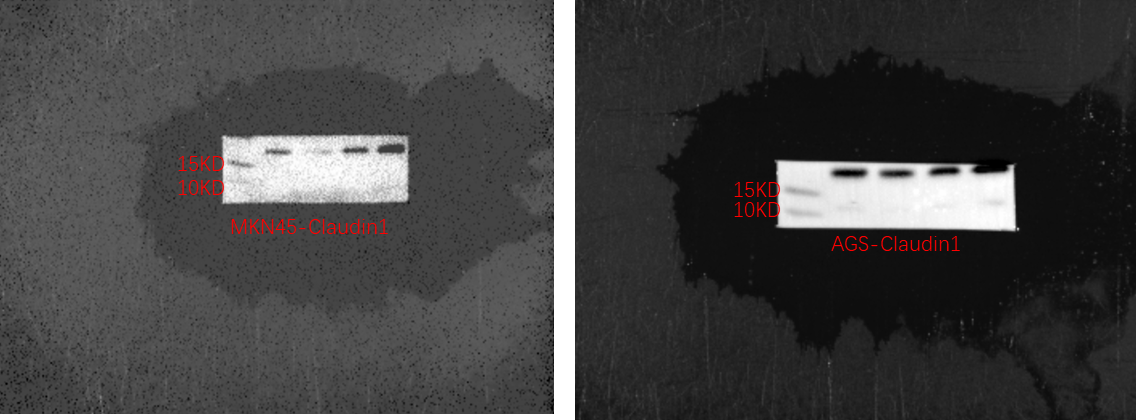


**Gastric cancer specimens**


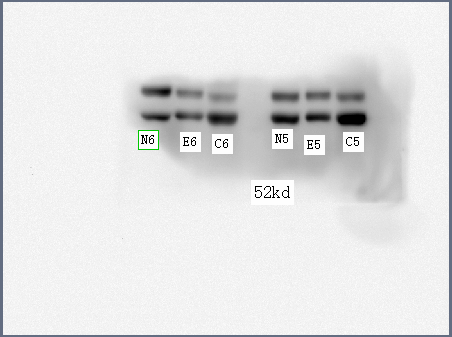

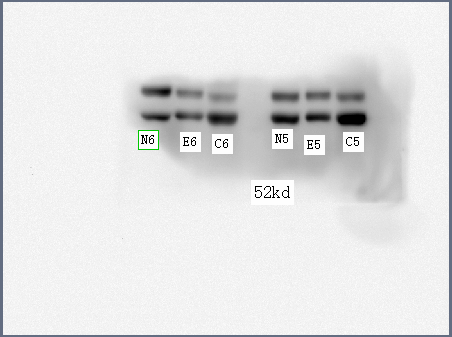

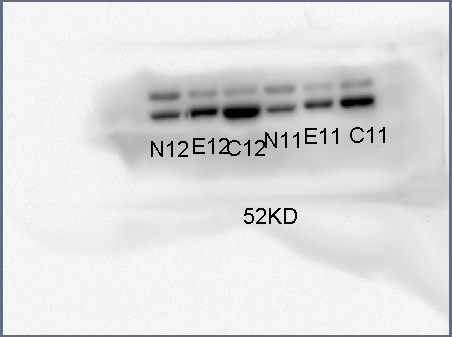

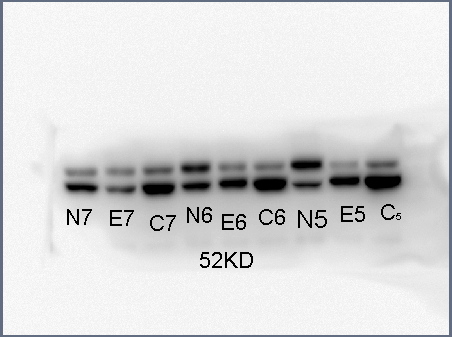

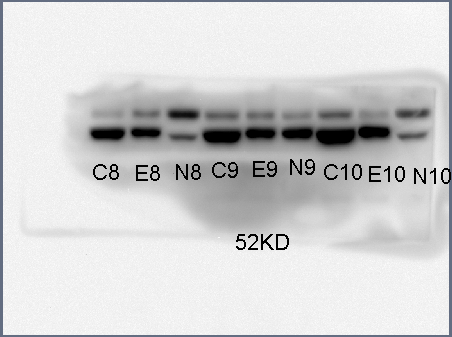

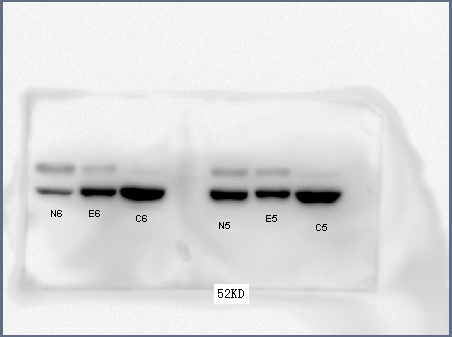

Supplement: Supplementary file 1 — Western blot [file 41420_2023_1767_MOESM1_ESM.docx]
